# Metagenomic Analysis of Distribution Characteristics and Driving Mechanisms of Antibiotic Resistance Genes, Virulence Factors, and Microbial Communities in Rice Seedling Cultivation Soils

**DOI:** 10.3390/microorganisms13112419

**Published:** 2025-10-22

**Authors:** Yu Zhong, Chanchan Wu, Zhipeng Yao, Xinyang Li, Hai Chi, Tao Wu, Xinglin Du

**Affiliations:** 1 Provincial Engineering Laboratory of Plant Genetic Improvement, College of Plant Science, Jilin University, Changchun 130062, China; zhongyu312@jlu.edu.cn (Y.Z.);; 2 Binzhou Academy of Agriculture Sciences, Binzhou 256600, China; 3 Key Laboratory of Protection and Utilization of Aquatic Germplasm Resource, Liaoning Ocean and Fisheries Science Research Institute, Dalian 116023, China

**Keywords:** antibiotic resistance genes (ARGs), virulence factors (VFs), microbial community, rice seedling cultivation soils

## Abstract

The extensive utilization of antibiotics in both healthcare and agricultural sectors has precipitated an exponential surge in antibiotic resistance genes (ARGs) and antibiotic-resistant bacteria (ARBs) within environmental matrices, thereby posing formidable threats to ecosystem stability and human health. Given soil’s pivotal role as a primary reservoir for ARGs and the inherent potential for these genes to translocate into agricultural produce, this study endeavors to evaluate the distribution patterns of ARGs and virulence factors (VFs) in soils designated for rice seedling cultivation. This study employed metagenomic sequencing to analyze antibiotic resistance genes (ARGs), virulence factors (VFs), and microbial communities in four rice seedling cultivation soils. The results revealed significant variations in microbial alpha diversity, community structure, ARGs, and VFs across soils, with multidrug resistance, glycopeptide resistance, and tetracycline resistance genes predominating. The inclusion of organic matter increased the complexity of the microbial network by increasing the levels of ARGs and VFs. Neutral community model analysis revealed that stochastic processes predominantly governed the assembly of microbial taxa, ARGs, and VFs, though ARGs were subject to stronger deterministic pressures. These communities were shaped by the pH, nitrogen, organic carbon content, electrical conductivity, and salinity of the soil. The core Actinobacteria genera acted as key vectors for ARGs and VFs dissemination. Our findings elucidate the complex interactions between microbes, ARGs, and VFs in cultivation soils and highlight that organic matter amendment, while enhancing fertility, can also increase the potential spread of microbial risk genes, underscoring the need for monitoring and managing ARGs and VFs in agricultural soils to mitigate public health risks.

## 1. Introduction

Antibiotics have been widely prescribed and unselectively used in the human healthcare sector and animal husbandry for decades, resulting in the vast expansion of antibiotic resistance genes (ARGs) in microbial communities [1,2]. The number of ARGs and antibiotic-resistant bacteria (ARBs) in the environment is growing exponentially, posing a significant danger to ecosystem security and human well-being [3,4]. Virulence factors (VFs) enable pathogenic bacteria to adhere to host cells (including those of plants, humans, and animals), initiate infection, and cause disease [5]. When ARGs and VFs coexist in the genome, they significantly enhance the potential risk to humans and animals [6].

Soil serves as a key reservoir for ARGs, with a wide variety and high diversity of ARGs discovered worldwide [7,8]. Multiple studies have verified that human practices (including agricultural operations such as irrigation, landfilling, and fertilization, especially organic fertilization) can significantly increase the concentration of ARGs in soil [9,10]. ARGs in agricultural soil can migrate to agricultural product tissues, such as stem leaves and fruits, through plant absorption and horizontal gene transfer, and then threaten human health through the food chain [11,12]. Therefore, assessing the distribution characteristics and interactions of ARGs and VFs during the cultivation process is of critical importance.

As a key global food crop, the development of efficient cultivation techniques for rice is of great significance for promoting its industrialization, achieving large-scale production, and enhancing labor productivity. Mechanized transplanting has emerged as the major technological approach. The key to successful mechanized rice transplanting is conquering the barrier of generating proper seedling growing media, allowing for the production of robust seedlings that can be mechanically transplanted. Currently, mechanized rice transplanting primarily relies on nutrient soil as a cultivation medium, with organic fertilizers (such as livestock manure) added to optimize seedling quality. Applying organic fertility produced by livestock farming reduces chemical fertilizer usage, maintains soil fertile, and boosts soil biodiversity [13]. However, the use of antibiotics in livestock farming may result in ARGs and ARBs-containing livestock manure, which, when applied to farmland soil, introduce such risk factors [14], posing potential risks of transmission through the food chain to consumers and jeopardizing health safety.

Given this, the current research focuses on a comprehensive analysis of the properties of antibiotic resistance gene clusters and virulence factor locations in rice seedling cultivation soil. Existing studies have revealed that soil physicochemical properties, including soil organic carbon (SOC), total nitrogen (TN), TN:TP ratio, and microbial biomass phosphorus (microbiomass-P), are closely related to the spatial mode and prevalence rate of ARGs or VFs in soil [15,16]. In addition, soil microbial communities harboring ARGs can significantly modify root-associated microbial communities via mutual interactions between the rhizosphere and soil, resulting in accelerated fitness of resistance genes in plant tissues [17]. Given that environmental heterogeneity is a core factor in determining bacterial community diversity and distribution patterns, changes in soil properties induced by organic fertilizer application are likely to have an indirect influence on ARGs distribution by reshaping the soil bacterial community structure [18]. Therefore, elucidating the complex intrinsic relationships between soil properties, microbial communities, and the distribution of ARGs and VFs in rice seedling cultivation soils has significant theoretical and practical implications.

In this study, we employed metagenomic sequencing technology to systematically analyze the distribution characteristics of soil microbial communities, ARGs, and VFs in four commonly used rice seedling cultivation soils available in the market. The specific research objectives encompass two aspects: first, to comprehensively investigate the current distribution status of microbial communities, ARGs, and VFs in rice seedling cultivation soils; and second, to explore the biological and non-biological driving factors influencing the composition and distribution of ARGs and VFs.

## 2. Materials and Methods

### 2.1. Soil Sampling

Four commonly used rice seedling cultivation soils (designated S1, S2, S3, and S4 for this study, collected from Jilin University farm, Jilin Province (45°3′5.64840″ N, 123°13′19.801200″ E)) were selected as experimental subjects. The organic content ranged from 28% to 40% among four cultivation soils. The total amount of nitrogen, phosphorus, and potassium ranged from 2% to 4%. Each type of rice seedling cultivation soil was sieved through a 2 mm mesh screen, placed in sealed plastic bags, and stored in a −80 °C freezer for subsequent molecular biological analysis, and each type of rice seedling cultivation soil had three replicate samples.

### 2.2. Analysis of Soil Properties

Soil pH was determined with a pH meter on suspensions prepared at a 1:2.5 (w v^−1^) soil-to-water ratio. Soil organic carbon (SOC) content was determined following the potassium dichromate oxidation-external heating procedure [19]. We quantified ammonium nitrogen (NH_4_^+^-N) levels using a microplate spectrophotometer (Thermo 1510, Multiskan GO, Thermo Fisher Scientific, Inc., Waltham, MA, USA). Electrical conductivity (EC), reflecting soil salinity, was measured with an EC meter. The alkali diffusion method served to analyze alkali-hydrolyzable nitrogen. Available phosphorus was extracted using 0.5 mol L^−1^ NaHCO_3_ solution, while available potassium was extracted with 1 mol L^−1^ ammonium acetate (NH_4_OAc) and quantified by flame photometry [19].

### 2.3. DNA Extraction, Metagenomics Sequencing and Data Analysis

Genomic DNA was extracted in triplicate from 0.5 g soil aliquots of each soil sample using the E.Z.N.A.^®^ Soil DNA Kit (Omega Bio-tek, Norcross, GA, USA). The DNA extracts from the three replicates for each sample were then pooled to create a composite DNA sample for subsequent metagenomic sequencing, ensuring representative coverage and mitigating the impact of small-scale heterogeneity. Shotgun metagenomic sequencing was performed using the Illumina Novaseq 6000 platform (Majorbio Bio-Pharm Technology Co., Ltd., Shanghai, China). Raw reads were processed with fastp (v0.20.0) for quality control, including adapter trimming and filtering. Specifically, reads were removed if they met any of these criteria: average quality score below Q20, presence of more than 3 ambiguous bases (N), length shorter than 50 bp, or alignment to common Illumina background sequences. Gene clustering was performed with CD-HIT (v4.6.1), with the longest sequence within each cluster designated as the representative for building a non-redundant gene catalog. The non-redundant gene catalog underwent BLASTP (v2.3.0) alignment against the NR database (v20200604). Taxonomic assignments were inferred from the associated taxonomic information within the NR database [20].

### 2.4. ARGs and VFs Analysis

ARGs annotation relied on the Comprehensive Antibiotic Resistance Database (CARD v3.0.9) and its Antibiotic Resistance Ontology (ARO) [21]. The non-redundant gene catalog was queried against CARD using BLASTP (v2.3.0), retaining only matches with E-values ≤ 1 × 10^−5^. For confident ARG assignment, hits needed to meet thresholds of ≥90% sequence identity and ≥25 amino acids aligned length. The lower aligned length threshold for ARGs (≥25 aa compared to ≥90 aa for VFs) was selected based on common practices in metagenomic studies focusing on ARGs [22], which aims to capture a broader diversity of ARG fragments while still maintaining reasonable confidence through high sequence identity. The stricter threshold for VFs (≥90 aa) was applied to increase the confidence in functional annotation due to the potential for shorter matches to non-virulence related domains. VFs identification involved BLAST alignment (E-value ≤ 1 × 10^−5^) of predicted open reading frames (ORFs) against the Virulence Factor Database (VFDB v2020.07.03). ORFs satisfying identity ≥ 90% and query coverage ≥ 90% were annotated as VFs [23]. Additionally, the likely bacterial hosts of ARGs and VFs were inferred through taxonomic annotation.

### 2.5. Statistical Analysis

Statistical analyses were performed to assess differences in soil properties, diversity indices, and the contributions of various factors to community composition. Physicochemical property variations across the four seedling substrates (S1–S4) were assessed via one-way ANOVA, supplemented by Duncan’s post hoc test for multiple comparisons (significance level α = 0.05). These statistical analyses were implemented using the agricolae package within R (v4.2.2). Data visualization (heatmaps, box plots, stacked bar plots) was conducted using the OmicStudio platform. Principal coordinates analysis (PCoA) and redundancy analysis (RDA) were performed employing the vegan package in R (v4.2.2) [24]. Procrustes analysis evaluated the concordance between soil microbial communities, ARGs, and VFs. The fit quality was quantified by the M^2^ statistic and associated *p*-values. The influence of stochastic dispersal processes on the assembly of soil microbial communities, ARGs, and VFs was evaluated using the neutral community model (NCM). Model fitting utilized the Hmisc and minpack.lm packages in R (v4.2.2) [25]. Co-occurrence networks were built considering soil microbial taxa, ARGs, and VFs that had a relative abundance exceeding 0.1%. Pairwise correlations were computed using the Hmisc package in R. To ensure robustness, only correlations with |r| ≥ 0.9 and Benjamini–Hochberg adjusted *p*-values < 0.01 were retained for network construction. Resulting networks were visualized in Gephi (v0.9.2). Unless otherwise specified, default parameters were used for the statistical packages mentioned below.

## 3. Results

### 3.1. Soil Properties

The soil properties varied substantially among the four rice seedling cultivation soils (S1, S2, S3, and S4) (Table 1). SOC and NH_4_^+^-N content were highest in S4, significantly exceeding those in the other treatments (*p* < 0.05). AN content was significantly higher in S1 and S4 than in S2 and S3. AP and AK contents peaked in S1, although AK in S3 did not differ significantly from that in S1. Soil pH reached its maximum in S4 and was significantly higher than that in the other treatments (S1, S2, S3). EC and salinity followed the order S1 > S4 > S2 ≈ S3. Collectively, S4 was distinguished by its high organic matter and ammonium nitrogen content, whereas S1 exhibited elevated salinity and high levels of available nutrients.

### 3.2. Microbial Diversity and Community Composition

Following quality control, each sample yielded 20.10–23.32 Gb of high-quality clean reads (Table S1). *De novo* assembly generated 1,001,958–2,183,001 contigs with N50 values of 756–1451 bp and N90 values of 364–408 bp. A total of 1,605,141–3,112,099 open reading frames (ORFs) were predicted from the data, with average lengths of 474–553 bp (Table S2). Bacteria dominated the sequences (99.1% of the total sequences) (Table S3).

Significant differences (*p* < 0.05) in the microbial alpha diversity indices were observed among the four nursery soils (Table 2). Species richness indices (Sobs, Ace, and Chao) were highest in S2 and significantly exceeded those in S4, S3, and S1. The Shannon diversity index was ordered as follows: S4 (6.18) > S1 (5.92) > S2 (5.17) > S3 (5.01). The Simpson index indicated that S2 exhibited the highest community evenness (0.034), significantly surpassing S3 (0.029), S1 (0.016), and S4 (0.010). The coverage value for all samples reached 1.00, confirming sufficient sequencing depth.

**Figure 1:**
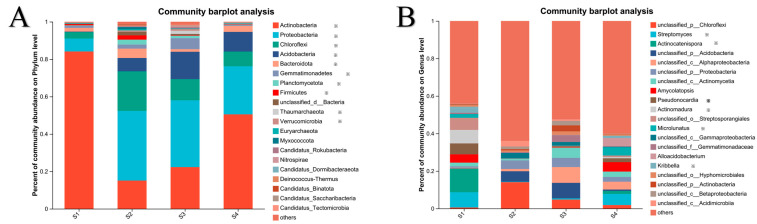
Compositional profile of microbial communities in four rice seedling cultivation soils at the phylum (**A**) and genus (**B**) level. Asterisk represents that the abundance differs significantly between groups.

Compositional analysis of microbial communities at the phylum and genus levels across the four rice seedling culture soil treatments (S1–S4) revealed significant structural changes in the dominant taxa among the treatments (Figure 1). Phylum-level analysis (Figure 1A) revealed Actinobacteria (relative abundance: 15.1–84.1%) and Proteobacteria (6.9–37.2%) as the dominant phyla in all treatments, with the following variations across treatments: S1 exhibited the highest relative abundance of Actinobacteria (84.1%); S2 was dominated by Proteobacteria (37.2%), followed by Chloroflexi; S3 showed co-dominance of Actinobacteria (22.3%) and Proteobacteria (35.7%), alongside significant enrichment of Acidobacteria; and S4 demonstrated absolute dominance of Proteobacteria (50.5%). Among the other phyla, Bacteroidota was more abundant in S2 and S4, whereas Gemmatimonadetes was more abundant in S2 and S3. Archaeal phyla (including Thaumarchaeota and Euryarchaeota) exhibited low relative abundance across all treatments. Additionally, Planctomycetota, Firmicutes, and Verrucomicrobia exhibited significant inter-treatment variation. Significant differences were also observed at the genus level (Figure 1B), with notable differential genera including *Streptomyces*, *Actinocatenispora*, *Pseudonocardia*, *Actinomadura*, *Microlunatus*, and *Kribbella*. Notably, the abundance of *Streptomyces* was significantly higher in S1 and S4 than in S2 and S3. Each treatment featured distinct dominant genera: S1 was characterized by *Actinocatenispora* and *Streptomyces*; S2 by unclassified_p_*Chloroflexi* and unclassified_p_*Acidobacteria*; S3 showed enrichment of unclassified_p_*Alphaproteobacteria* and unclassified_p_*Acidobacteria*; and S4 displayed the prominence of *Amycolatopsis* alongside *Streptomyces*. Remarkably, unclassified microorganisms (“others”) accounted for 44.1–64.0% across treatments, indicating substantial uncultured microbial diversity with undefined taxonomic and functional characteristics.

### 3.3. Abundance and Composition of ARGs and VFs

**Figure 2:**
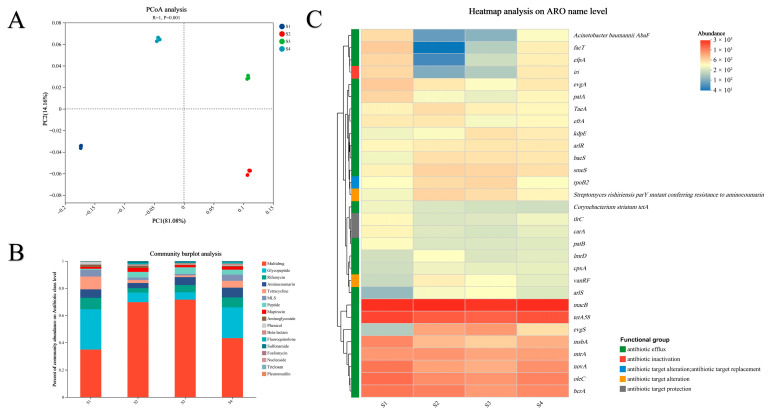
(**A**) Principal coordinate analysis (PCoA) of antibiotic resistance genes (ARGs) across four rice seedling cultivation soils; (**B**) Relative abundance bar plot of ARG classes; (**C**) Heatmap of total abundance of ARG subtypes.

Principal coordinate analysis (PCoA) revealed significant differences in ARGs composition among the four rice seedling cultivation soils (S1–S4) (Figure 2A; *p* = 0.001), with subsequent analyses at the resistance class and gene subtype levels demonstrating pronounced inter-treatment heterogeneity (Figure 2B,C). Among the 17 identified resistance classes, multidrug (34.84–71.57%), glycopeptide (5.38–29.67%), rifamycin (3.28–8.40%), aminocoumarin (3.77–7.16%), tetracycline (1.20–9.39%), and MLS (1.13–4.90%) constituted the core ARGs components (Table S4). Analysis of the top 30 abundant ARGs subtypes showed differing dominant profiles across treatments: S1 was characterized by *macB* (2981.8 TPM), *tetA*(58) (2420.5 TPM), *oleC* (1659.2 TPM), *bcrA* (1506.6 TPM), and *novA* (1505.5 TPM); S2 was overwhelmingly dominated by *macB* (3081.8 TPM); S3 exhibited the highest abundances of *macB* (2825.7 TPM) and *tetA(58)* (1846.2 TPM); and S4 showed significant enrichment of *macB* (3054.0 TPM), *tetA(58)* (2119.6 TPM), *oleC* (1315.8 TPM), and *bcrA* (1218.9 TPM) (Table S5). Notably, the *macB* sub-type maintained consistently high abundance across all treatments, indicating its ubiquity within the seedling cultivation soil resistome.

**Figure 3:**
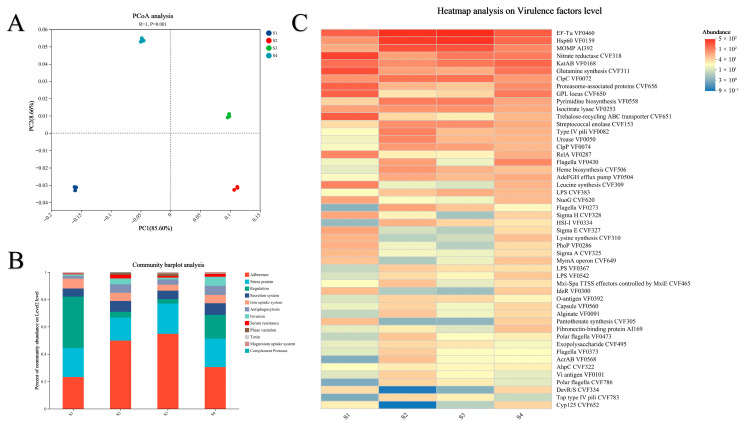
(**A**) Principal coordinates analysis (PCoA) of virulence factors (VFs) in four rice seedling cultivation soils; (**B**) Bar plot of relative abundance of VF functional categories; (**C**) Heatmap of VFs.

PCoA revealed significant compositional differences in VFs among the four rice seedling cultivation soils (S1, S2, S3, and S4) (*p* = 0.001; Figure 3A). Functional category analysis (Figure 3B) demonstrated that adherence (407.636–1419.436 TPM), stress protein (372.834–509.479 TPM), regulation (78.239–657.257 TPM), secretion system (104.193–227.743 TPM), and iron uptake system (102.766–167.211 TPM) constituted the predominant categories shared across all treatments (Table S6). Analysis of the top 50 abundant virulence factors (Figure 3C) further revealed treatment-specific enrichment patterns: S1 featured significant overexpression of *Mycobacterium avium* subsp. (Nitrate reductase, 387.441 TPM), *Mycobacterium tuberculosis* str. (glutamine synthesis, 318.496 TPM), *Mycobacterium* sp. (trehalose-recycling ABC transporter, 256.467 TPM), *Francisella tularensis* subsp. (EF-Tu, 244.905 TPM), *Mycobacterium ulcerans* (GPL locus, 243.047 TPM), and *Mycobacterium smegmatis* strain (Proteasome-associated proteins, 242.550 TPM), and S2 was primarily enriched in *Francisella tularensis* subsp. (EF-Tu, 502.751 TPM), *Legionella pneumophila* subsp. (Hsp60, 421.799 TPM), and *Chlamydia trachomatis* (MOMP, 311.560 TPM); S3 was dominated by *Francisella tularensis* subsp. (EF-Tu, 522.426 TPM), *Legionella pneumophila* subsp. (Hsp60, 453.870 TPM), *Chlamydia trachomatis* (MOMP, 295.112 TPM), and *Listeria monocytogenes* (ClpC, 195.585 TPM); while S4 showed significant enrichment of *Francisella tularensis* subsp. (EF-Tu, 266.135 TPM), *Legionella pneumophila* subsp. (KatAB, 228.424 TPM and Hsp60, 222.053 TPM), and *Mycobacterium tuberculosis* strain (glutamine synthesis, 185.891 TPM) (Table S7).

### 3.4. Assembly Processes and Environmental Drivers of Microbial Taxa, ARGs and VFs

**Figure 4:**
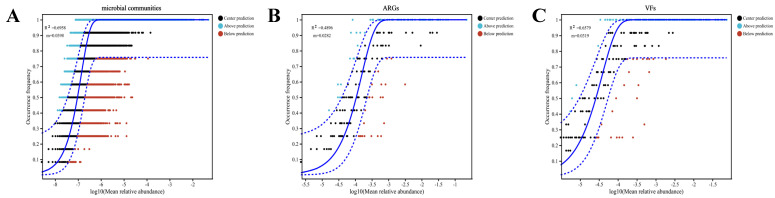

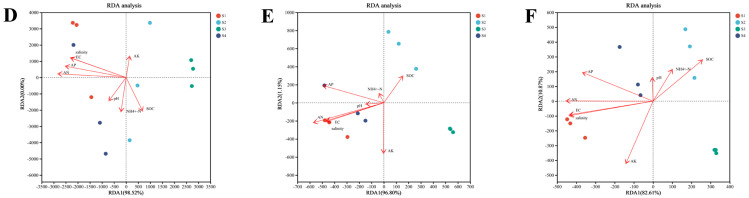
(**A**) The Neutral community model (NCM) for soil microbial communities in four rice seedling cultivation soils; (**B**) The neutral community model (NCM) for ARGs in four rice seedling cultivation soils; (**C**) Neutral community model (NCM) for soil microbial communities VFs in four rice seedling cultivation soils. The *R*^2^ value indicates the goodness-of-fit to the model, and “*m*” represents the migration rate. Solid blue lines denote the optimal model fits, with dashed lines showing the 95% confidence intervals around the model predictions. Genes deviating from predictions occurring more or less frequently than expected are highlighted in distinct colors. (**D**) Redundancy analysis (RDA) showing correlations between soil properties and soil microbial communities across four rice seedling cultivation soils; (**E**) Redundancy analysis (RDA) showing correlations between soil properties and ARGs across four rice seedling cultivation soils; (**F**) Redundancy analysis (RDA) showing correlations between soil properties and VFs across four rice seedling cultivation soils. AN, alkaline hydrolyzable nitrogen; AK, available potassium; AP, available phosphorus; NH_4_^+^-N, ammonium nitrogen; SOC, soil organic carbon; pH, soil pH; EC, electrical conductivity; Salinity, soil salinity.

The Neutral Community Model (NCM) analysis demonstrated that stochastic factors strongly influenced the assembly processes of microbial taxa, ARGs, and VFs (Figure 4A–C). Microbial communities exhibited the highest stochastic process fit (R^2^ = 0.6958) and a migration rate (*m*) of 0.0398, indicating that environmental dispersal is the primary mechanism driving their assembly (Figure 4A). In contrast, ARGs showed significantly lower stochasticity (R^2^ = 0.4896) and migration rate (*m* = 0.0282) than microbial communities, suggesting stronger niche selection constraints on their assembly (Figure 4B). VFs displayed intermediate stochasticity (R^2^ = 0.6579, *m* = 0.0319), with a dispersal capacity positioned between microbial communities and ARGs (Figure 4C). Collectively, these findings indicate that stochastic dispersal processes exert a relatively stronger influence on microbial community structure, whereas ARGs assembly is concurrently subject to significant deterministic pressures. The migration pattern of VFs further suggests that their dispersal may be mediated by host microbial activities.

Soil physicochemical properties were significantly correlated with the composition of microbial communities, ARGs, and VFs. Redundancy analysis (RDA) revealed that RDA1 (98.52%) and RDA2 (0.00%) accounted for 98.52% of the variation in microbial communities (Figure 4D). RDA1 (96.80%) and RDA2 (1.15%) accounted for 97.95% of the ARG variance (Figure 4E). RDA1 (82.61%) and RDA2 (10.87%) accounted for 93.48% of the variation in the VFs. These high percentages indicate that the measured environmental variables explain the vast majority of the observable variation in the microbial communities, ARGs, and VFs among the four soils. In the RDA plots (Figure 4D–F), the positions of the samples (S1–S4) reflect their compositional similarities, while the direction and length of the environmental factor arrows indicate the strength and direction of their correlation with the community and gene profiles. Alkali-hydrolyzable nitrogen (AN), available phosphorus (AP), electrical conductivity (EC), and salinity were identified as key environmental factors driving the composition of microbial communities, ARGs, and VFs (Table S8).

### 3.5. Relationships Between Microbial Taxa, ARGs and VFs

**Figure 5:**
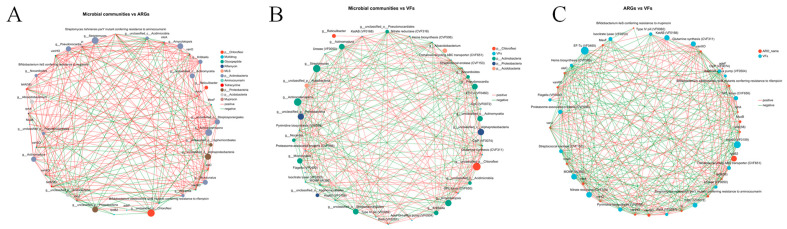
(**A**) Interaction networks of microbial communities and antibiotic resistance genes (ARGs) across four rice seedling cultivation soils; (**B**) Interaction networks of microbial communities and virulence factors (VF) across four rice seedling cultivation soils; (**C**) Interaction networks of antibiotic resistance genes (ARGs) and virulence factors (VFs) across four rice seedling cultivation soils.

Microbial correlation network analysis of nursery soils revealed intricate and robust interactions between microbial taxa, ARGs, and VFs (Figure 5). Within ARG-VF co-occurrence networks, core virulence factors (e.g., MOMP, GPL locus, heme biosynthesis) and key resistance genes (e.g., *mtrA, vanXO*) exhibited strong connectedness and betweenness centrality, acting as pivotal hubs driving their synergistic relationships. Actinobacteria (particularly the genera *Actinomadura, Kribbella*, and *Streptomyces*) occupied central positions in both microbe-VF and microbe-ARG networks, with their elevated connectivity and betweenness centrality indicating their critical role as vectors for ARG and VF spread. The exceptionally high betweenness centrality of unclassified_p_*Acidobacteria* in microbe-ARG networks further underscored their bridging function in dispersal of resistance genes. At the functional gene level, the prominent betweenness centrality of the virulence factor KatAB in microbe-VF networks and the hub status of the resistance gene *novA* in microbe-ARG networks demonstrate that these essential genes dominate cross-network interactions. Furthermore, the strong association between *Streptomyces* and multiple ARGs corroborates its role as a resistance reservoir, whereas low-connectivity taxa (e.g., *Bifidobacterium* spp. harboring the *ileS* gene) may indicate strain-specific resistance mechanisms (Table S9). Conclusively, core Actinobacteria (especially *Streptomyces*) and unclassified_p_*Acidobacteria* drive the co-dissemination of ARGs and VFs, with pivotal genes such as KatAB and *novA* serving as network hubs to shape the complex microbe-resistance-virulence interactome in nursery soils.

## 4. Discussion

Microbial populations in cultivated soil are closely linked to antibiotic resistance genes, which have major consequences for soil health, food safety, and human well-being [26,27]. Numerous studies have demonstrated how organic fertilizers can boost the diversity and structure of soil microbial communities [28,29]. The addition of organic matter increases the organic carbon and available nitrogen content in the soil [29], creating a more favorable nutritional environment for microorganisms. Additionally, organic fertilizers can effectively regulate soil acidification and create a more suitable environment for soil microorganisms to thrive [30,31]. However, despite the numerous benefits of livestock-derived organic fertilizers for soil, their application also poses risks of antibiotic, pathogenic factors, and pathogen contamination [32]. This study found that SOC significantly altered the composition of ARGs and increased their abundance, consistent with previous research findings [33]. Antibiotics, ARBs, and ARGs are abundant in poultry manure, and if applied to fields, these compounds are highly likely to spread into the surrounding ecosystems [33].

The four seedling substrates mainly enriched resistance genes associated with multidrug resistance, glycopeptides, rifamycins, sulfonamides, tetracyclines, and MLS-type antibiotics. Previous studies have confirmed that spreading manure in agricultural ecosystems introduces additional antibiotics [34,35,36]. Further analysis revealed that among the major microbial risk genes and antibiotic resistance gene subtypes significantly enriched in organic matter, the *macB* subtype associated with MLS remained abundant across all treatments, indicating its widespread presence in the resistance genome of seedling cultivation soils. Generic genes highly related to antibiotic efflux mechanisms include aminocoumarin (*novA*), tetracycline (*tetA(58)*), MLS (*macB* and *oleC*), and peptide *(bcrA)* [37]. Additionally, all four seedling substrates had considerably more virulence factors related to adhesion, stress proteins, regulation, secretion systems, and iron uptake systems. Bacterial pathogens adhere to host cells by producing protein or polysaccharide surface layers, and specialized enzymes invade host cells and tissues [37]. Stress proteins play a key role in the persistence and survival of intracellular pathogens. The significant abundance of these virulence factors in seedling substrates implies that bacterial pathogens may have better colonization and survival capabilities in these environments. VFs are predominantly impacted by dietary factors found in sediments [38]. Soborg et al. [39] confirmed that the presence of virulence genes in the environment is rarely induced by clinically relevant bacteria, suggesting that these genes may have an environmental origin. Their findings also indicated that higher pH values corresponded to lower virulence factor dispersion. Based on these findings, the addition of organic fertilizer to the four types of seedling substrates increased the levels of certain ARGs and VFs, posing a potential serious threat to community health.

Microorganism risk is associated not only to microbial community diversity, abundance, ARGs, and VFs, but also to patterns of coexistence in the same ecological niche [40,41]. Significant connections were discovered between microbial communities, ARGs, and VFs using Procrustes and network analysis [40,41]. Our results showed that elevated organic matter abundance promotes network connectivity [42], possibly facilitating the consilience of microbial taxa, antibiotic resistance genes (ARGs), and VFs, thus aggravating microbial pollution risks [35]. This study also indicates that higher organic matter content promotes favorable interactions between microbial communities-ARGs, microbial communities-VF, and ARGs-VF, lending significant support to the previous conclusions [34,37]. In the network structure, actinomycetes (especially the genera *Actinomadura, Kribbella,* and *Streptomyces*) occupy central positions in the microbial-VF and microbial-ARG networks. Their strong connectivity and intermediary centrality suggest that microbial communities may harbor multiple ARG subtypes and VFs, acting as important carriers for the transmission of these genes and factors, and playing a key role in the proliferation and spread of ARGs and VFs in soil [43].

Both deterministic and stochastic processes play important roles in the assembly of soil microbial communities, ARGs and VFs [44,45]. The results of this study support the dominant role of stochastic processes in the assembly of soil microbial communities, ARGs and VFs. Specifically, organic matter reduces resource competition among soil microbial communities by increasing resource availability, leading to the dominance of randomness in soil microbial community assembly. To some extent, environmental stress regulates ARGs and VFs formation in a similar way that resources regulate soil microbial community assembly [23]. This study found that soil microbial communities, ARGs, and VFs were significantly affected by factors such as soil pH, nitrogen content, and SOC, which is consistent with previous research findings [35,45,46,47]. Prior researchers have demonstrated that soil pH notably influences on the adsorption and desorption processes of ARGs; however, organic carbon, total nitrogen, and available potassium content modify the distribution structure of soil ARGs [35]. Yang et al. revealed that soil electrical conductivity is inversely correlated with the absolute abundance of genes linked to the anti-phagocytic system, secretion system, and toxins [21]. Since both ARGs and VFs are located in potential microbial hosts, the strong link between soil physicochemical properties, enzyme activity, and resistance groups may be mediated by the soil microbial community structure [48]. This study utilized metagenomic sequencing technology to perform a comprehensive analysis of ARGs, VFs, and microbial communities in the soil of rice seedling. The findings revealed significant differences in microbial diversity and the abundance of ARGs and VFs across different soil types, highlighting the impact of organic matter addition on enhancing microbial network complexity and connectivity in soil.

The widespread presence of multidrug resistance, glycopeptide, and tetracycline resistance genes underscores the potential risks of agricultural soil contamination to public health. Notably, random processes shape the assembly of soil microbial communities, ARGs, and VFs, and are influenced by key soil properties such as pH, nitrogen content, and organic carbon. Core actinomycete genera, particularly *Actinomadura, Kribbella,* and *Streptomyces*, were identified as primary carriers of ARGs and VFs, underscoring their important role in soil microbial ecology and potential implications for public health in the future. These findings highlight the importance of monitoring and managing ARGs and VFs in agricultural soils to mitigate the spread of antibiotic resistance and protect human health. Future research should focus on developing strategies to reduce the introduction and spread of resistant elements in agricultural ecosystems. Potential interventions include the pre-treatment of manure through thermal, composting, or anaerobic digestion processes to diminish resistant elements prior to land application; the implementation of more stringent regulations on antibiotic usage in livestock farming to curtail the prevalence of resistant bacteria in manure; the exploration of alternative, non-animal-derived organic amendments, such as plant-based composts, to decrease reliance on contaminated manure; the optimization of soil management practices, including the modulation of pH, organic carbon, and nitrogen levels, to suppress ARG proliferation; and the utilization of bioremediation techniques employing ARG-degrading microorganisms or phages to specifically target resistant bacteria in agricultural soils. These proposed solutions can effectively complement the authors’ advocacy for research into mitigation strategies by offering practical and actionable measures to curtail ARG transmission within agricultural ecosystems.

## 5. Conclusions

This study provides valuable insights into the distribution and driving mechanisms of ARGs, VFs, and microbial communities in rice seedling cultivation soil. The findings emphasize the importance of integrated soil management strategies that balance between agricultural productivity with environmental and public health concerns. Future research should focus on developing effective interventions to prevent the introduction and spread of antibiotic resistance in agriculture.

## Figures and Tables

**Table 1:** Basic properties of four rice seedling cultivation soils.

Treatments	S1	S2	S3	S4
AN ^1^ (mg/kg)	112.85 ± 2.92 a	59.72 ± 1.30 c	53.70 ± 2.83 c	103.08 ± 2.36 b
AK ^2^ (mg/kg)	247.05 ± 2.24 a	177.08 ± 3.06 c	246.34 ± 0.97 a	209.91 ± 0.60 b
AP ^3^ (mg/kg)	76.27 ± 2.05 a	64.45 ± 3.0 b	32.65 ± 2.58 d	54.07 ± 2.79 c
NH4+-N ^4^ (mg/kg)	143.28 ± 3.22 c	219.19 ± 0.74 b	214.63 ± 4.00 b	340.65 ± 4.37 a
SOC ^5^ (g/kg)	132.46 ± 4.21 d	435.69 ± 11.00 b	347.30 ± 4.93 c	512.36 ± 0.88 a
pH ^6^	6.31 ± 0.18 c	6.45 ± 0.04 b	6.55 ± 0.11 b	7.27 ± 0.14 a
EC ^7^ (μS/cm)	1922.67 ± 143.18 a	707.00 ± 16.82 c	497.33 ± 6.43 d	847.33 ± 18.34 b
Salinity ^8^ (g/kg)	1.02 ± 0.06 a	0.36 ± 0.01 c	0.26 ± 0.01 d	0.45 ± 0.02 b

The data are presented as mean ± standard deviation (SD). Values within a row followed by the same lowercase letter are not significantly different according to Duncan’s multiple range test (*p* > 0.05). ^1^ alkaline hydrolyzable nitrogen; ^2^ available potassium; ^3^ available phosphorus; ^4^ ammonium nitrogen; ^5^ soil organic carbon; ^6^ soil pH; ^7^ electrical conductivity; ^8^ soil salinity.

**Table 2:** Alpha diversity indices of four rice seedling cultivation soil.

Samples	Sobs	Ace	Chao	Shannon	Simpson	Coverage
S1	16,859.33 ± 52.70 d	16,859.33 ± 52.70 d	16,859.33 ± 52.70 d	5.92 ± 0.01 b	0.016 ± 0.0006 c	1
S2	19,107.00 ± 106.93 a	19,107.00 ± 106.93 a	19,107.00 ± 106.93 a	5.17 ± 0.03 c	0.034 ± 0.0008 a	1
S3	17,922.33 ± 5.03 c	17,922.33 ± 5.03 c	17,922.33 ± 5.03 c	5.01 ± 0.02 d	0.029 ± 0.0004 b	1
S4	18,498.00 ± 120.62 b	18,498.00 ± 120.62 b	18,498.00 ± 120.62 b	6.18 ± 0.01 a	0.010 ± 0.0002 d	1

The data are presented as mean ± standard deviation (SD). Values assigned the same letter indicate no significant difference between groups (*p* > 0.05).

## Data Availability

The original contributions presented in this study are included in the article/Supplementary Material. Further inquiries can be directed to the corresponding author.

## Supplementary Materials

The following supporting information can be downloaded at: https://www.mdpi.com/article/10.3390/microorganisms13112419/s1, Table S1: Overview of sequences merge-ability and number of annotations made for shotgun-metagenome datasets of each sample in four rice seedling cultivation soils; Table S2: Overview of genes catalog assembly to open reading frames (ORFs) of each sample in four rice seedling cultivation soils; Table S3: Relative abundance (%) of identified kingdom in four rice seedling cultivation soils; Table S4: Relative abundance (%) of antibiotic class in four rice seedling cultivation soils; Table S5: Abundance (TPM) of top 30 antibiotics resistance genes (ARGs) in four rice seedling cultivation soils; Table S6: Abundance (TPM) of virulence factors (VFs) types in four rice seedling cultivation soils; Table S7: Abundance (TPM) of top 50 virulence factors (VFs) in four rice seedling cultivation soils; Table S8: Effects of soil properties on soil microbial communities, antibiotics resistance genes (ARGs) and virulence factors (VFs); Table S9: Topological parameters of Microbial-ARG-VF interaction networks across four rice seedling cultivation soils.
