# Supplementary material for: Metagenomic Analysis of Distribution Characteristics and Driving Mechanisms of Antibiotic Resistance Genes, Virulence Factors, and Microbial Communities in Rice Seedling Cultivation Soils
Source: Microorganisms. 2025 Oct 22;13(11):2419. doi: 10.3390/microorganisms13112419 (PMC12654301; doi:10.3390/microorganisms13112419)
Supplement: Supplementary file 1 [file microorganisms-13-02419-s001.zip › microorganisms-3842492-supplementary.pdf]

Contens

Supplementary tables 2

Table S1 Overview of sequences merge-ability and number of annotations made for shotgun-metagenome datasets of each sample in four rice seedling cultivation soils....... 2

Table S2 Overview of genes catalog assembly to open reading frames (ORFs) of each sample in four rice seedling cultivation soils. 3

Table S3 Relative abundance (%) of identified kingdom in four rice seedling cultivation soils.. 4

Table S4 Relative abundance (%) of antibiotic class in four rice seedling cultivation soils.. 5

Table S5 Abundance (TPM) of top 30 antibiotics resistance genes (ARGs) in four rice seedling cultivation soils. 6

Table S6 Abundance (TPM) of virulence factors (VFs) types in four rice seedling cultivation soils.. 8

Table S7 Abundance (TPM) of top 50 virulence factors (VFs) in four rice seedling cultivation soils.. 9

Table S8 Effects of soil properties on soil microbial communities, antibiotics resistance genes (ARGs) and virulence factors (VFs)..11

Table S9 Topological parameters ofMicrobial-ARG-VF interaction networks across four rice seedling cultivation soils. 12

Supplementary Tables

Table S1 Overview of sequences merge-ability and number of annotations made for shotgun-metagenome datasets of each sample in four rice seedling cultivation soils.

Sample	Raw reads	Raw bases (Gp)	Clean reads	Clean bases (Gp)	Contigs	Contigs bases (Bp)	N50 length (Bp)	N90 length (Bp)
S1-1	153909184	23.24	147184930	21.94	1044274	1060707731	1451	408
S1-2	152248104	22.99	145390238	21.65	1057189	1063280710	1424	406
S1-3	141737602	21.40	135327870	20.13	1001958	1010494315	1436	407
S2-1	161055702	24.32	154448560	23.05	2183001	1756269127	889	376
S2-2	152866364	23.08	146585036	21.89	2039814	1668180650	913	378
S2-3	144730120	21.85	138995716	20.75	1992213	1577552275	869	373
S3-1	147123386	22.22	141281178	21.09	1739733	1285139773	772	366
S3-2	148346280	22.40	142474128	21.28	1786321	1301014481	756	364
S3-3	152405290	23.01	146433870	21.87	1835519	1335950080	758	364
S4-1	162819580	24.59	156139172	23.32	1449898	1350782100	1213	395
S4-2	140318002	21.19	134738842	20.10	1302254	1204839083	1199	394
S4-3	145502538	21.97	140180048	20.92	1382604	1268000631	1172	392

Table S2 Overview of genes catalog assembly to open reading frames (ORFs) of each sample in four rice seedling cultivation soils.

Sample	Total length (Bp)	Average length (Bp)	Open reading frames (ORFs)
S1-1	928986045	552.61	1681083
S1-2	929835387	549.68	1691609
S1-3	887186679	552.72	1605141
S2-1	1581756870	508.26	3112099
S2-2	1501120578	512.44	2929332
S2-3	1415940852	503.67	2811244
S3-1	1151217543	477.91	2408836
S3-2	1165696161	474.63	2456034
S3-3	1196039688	474.22	2522115
S4-1	1195571061	533.65	2240366
S4-2	1068134286	531.9	2008139
S4-3	1124168952	530.08	2120743

Table S3 Relative abundance (%) of identified kingdom in four rice seedling cultivation soils.

Kingdom	S1	S2	S3	S4	Mean
Archaea	0.255%	0.763%	1.868%	0.224%	0.778%
Bacteria	99.587%	99.212%	98.094%	99.644%	99.134%
Eukaryota	0.152%	0.012%	0.010%	0.115%	0.072%
Viruses	0.003%	0.005%	0.018%	0.015%	0.010%
Unknown	0.003%	0.008%	0.009%	0.003%	0.006%

Values are means (n = 3).

Table S4 Relative abundance (%) of antibiotic class in four rice seedling cultivation soils.

Antibiotic class	S1	S2	S3	S4
Multidrug	34.84	69.67	71.57	43.21
Glycopeptide	29.67	7.08	5.38	22.70
Rifamycin	8.40	3.28	5.35	7.29
Aminocoumarin	6.25	3.77	5.87	7.16
Tetracycline	9.39	2.15	1.20	5.03
MLS	4.90	1.95	1.13	4.67
Peptide	0.84	4.11	4.89	3.55
Mupirocin	1.33	2.91	1.52	2.29
Aminoglycoside	1.76	1.64	0.33	0.64
Phenicol	1.87	0.23	0.14	0.50
Beta-lactam	0.32	0.73	1.03	1.10
Fluoroquinolone	0.18	0.93	0.45	1.09
Sulfonamide	0.12	1.41	0.99	0.50
Fosfomycin	0.01	0.03	0.03	0.23
Nucleoside	0.10	0.01	0.00	0.01
Triclosan	0.00	0.05	0.14	0.02
Pleuromutilin	0.00	0.05	0.00	0.00

Table S5 Abundance (TPM) of top 30 antibiotics resistance genes (ARGs) in four rice seedling cultivation soils.

Antibiotic subtype	Antibiotic class	S1	S2	S3	S4
macB	MLS	2981.782	3081.787	2825.668	3053.974
tetA(58)	Tetracycline	2420.522	1953.643	1846.225	2119.604
oleC	MLS	1659.226	1201.422	1084.853	1315.772
bcrA	Peptide	1506.619	1363.009	1028.342	1218.932
novA	Aminocoumarin	1505.483	965.628	855.130	1151.197
mtrA	Multidrug	1070.984	1104.503	935.460	989.681
msbA	Multidrug	1256.336	732.732	603.075	835.316
evgS	Multidrug	163.026	931.721	1048.167	508.331
smeS	Multidrug	412.948	581.870	556.596	499.293
evgA	Multidrug	638.948	459.958	348.649	460.446
rpoB2	Multidrug	367.135	522.406	564.114	355.368
Streptomyces rishiriensis parY mutant conferring resistance to aminocoumarin	Aminocoumarin	321.024	564.948	517.549	397.512
arlR	Multidrug	393.453	446.409	470.210	463.270
baeS	Multidrug	315.215	501.664	485.497	463.794
TaeA	Pleuromutilin	407.232	518.414	414.721	389.225
efrA	Multidrug	447.763	465.083	333.283	388.775
kdpE	Aminoglycoside	310.705	343.317	490.216	440.869
patA	Fluoroquinolone	562.947	334.662	285.394	399.156
vanRF	Glycopeptide	201.710	429.773	361.698	272.065

facT	Elfamycin	625.590	42.838	167.222	426.149
efpA	Multidrug	552.556	56.832	215.371	424.391
iri	Rifamycin	542.189	97.033	159.578	449.838
tlrC	Multidrug	387.460	244.327	251.092	308.927
carA	Multidrug	404.621	253.831	236.772	275.158
patB	Fluoroquinolone	375.316	250.137	247.751	261.735
lmrD	Multidrug	224.726	364.924	246.556	268.589
cpxA	Aminoglycoside	217.699	307.434	283.189	282.042
arlS	Multidrug	123.146	334.582	359.784	255.696
Acinetobacter baumannii AbaF	Fosfomycin	521.317	76.623	108.506	347.331
Corynebacterium striatum tetA	Aminoglycoside	309.352	243.533	225.424	217.983

Table S6 Abundance (TPM) of virulence factors (VFs) types in four rice seedling cultivation soils.

VFs types	S1	S2	S3	S4
Adherence	407.636	1419.436	1268.099	674.247
Stress protein	372.834	482.213	509.479	462.916
Regulation	657.257	118.481	78.239	384.519
Secretion system	104.193	227.743	142.356	189.560
Iron uptake system	128.359	167.211	102.766	132.506
Antiphagocytosis	34.353	181.544	87.118	151.391
Invasion	19.128	119.276	31.522	144.682
Serum resistance	10.536	74.878	28.387	44.330
Phase variation	2.750	47.415	42.590	6.141
Toxin	12.951	7.659	14.289	10.136
Magnesium uptake system	3.518	0.018	7.600	13.742
Complement Protease	0.028	1.831	0.056	0.361

Table S7 Abundance (TPM) of top 50 virulence factors (VFs) in four rice seedling cultivation soils.

VFs	Genus	Species	S1	S2	S3	S4
EF-Tu	Francisella	Francisella tularensis subsp.	244.905	502.751	522.426	266.135
Hsp60	Legionella	Legionella pneumophila subsp.	107.626	421.799	453.870	222.053
MOMP	Chlamydia	Chlamydia trachomatis	81.182	311.560	295.112	137.137
Nitrate reductase	Mycobacterium	Mycobacterium avium subsp.	387.441	94.007	170.950	168.385
KatAB	Legionella	Legionella pneumophila subsp.	201.619	124.530	173.472	228.424
Glutamine synthesis	Mycobacterium	Mycobacterium tuberculosis str.	318.496	91.785	79.850	185.891
ClpC	Listeria	Listeria monocytogenes	111.468	180.455	195.585	104.445
Proteasome-associated proteins	Mycobacterium	Mycobacterium smegmatis str	242.550	67.503	51.109	127.224
GPL locus	Mycobacterium	Mycobacterium ulcerans	243.047	33.457	40.604	168.297
Pyrimidine biosynthesis	Francisella	Francisella tularensis subsp.	42.934	163.585	161.540	85.640
Isocitrate lyase	Mycobacterium	Mycobacterium tuberculosis	92.605	109.142	118.448	83.504
Trehalose-recycling ABC transporter	Mycobacterium	Mycobacterium sp.	256.467	40.047	29.221	66.809
Streptococcal enolase	Streptococcus	Streptococcus gordonii str.	38.498	122.350	123.803	80.285
Type IV pili	Yersinia	Yersinia enterocolitica subsp.	21.541	150.896	60.363	71.260
Urease	Helicobacter	Helicobacter pylori	17.059	137.061	65.022	66.946
ClpP	Listeria	Listeria monocytogenes	20.186	103.055	93.026	64.721
RelA	Mycobacterium	Mycobacterium tuberculosis	143.277	27.309	26.157	75.922
Flagella	Helicobacter	Helicobacter pylori	17.539	98.525	16.792	138.653
Heme biosynthesis	Haemophilus	Haemophilus somnus	14.668	109.745	70.623	56.350
AdeFGH efflux pump	Acinetobacter	Acinetobacter baumannii	20.322	82.883	61.878	81.977
Leucine synthesis	Mycobacterium	Mycobacterium gilvum	147.882	16.373	12.331	70.166
LPS	Brucella	Brucella melitensis bv	21.016	52.692	78.069	63.281
NuoG	Mycobacterium	Mycobacterium gilvum	88.985	20.586	17.689	63.228
Flagella	Helicobacter	Helicobacter pylori	3.675	90.305	52.795	23.564
Sigma H	Mycobacterium	Mycobacterium sp.	88.591	26.019	5.667	42.917
HSI-I	Pseudomonas	Pseudomonas aeruginosa	4.428	71.419	41.985	31.904
Sigma E	Mycobacterium	Mycobacterium intracellulare	91.354	11.093	7.148	37.554
Lysine synthesis	Mycobacterium	Mycobacterium gilvum	68.653	7.561	10.137	58.980
PhoP	Mycobacterium	Mycobacterium tuberculosis	74.694	17.283	7.441	38.626
Sigma A	Mycobacterium	Mycobacterium intracellulare str.	63.695	18.696	16.618	36.501
MymA operon	Mycobacterium	Mycobacterium gilvum	54.936	6.432	16.550	55.298
LPS	Brucella	Brucella melitensis bv	8.997	48.297	36.615	34.505
LPS	Francisella	Francisella tularensis subsp.	11.927	38.180	24.626	52.565
Mxi-Spa TTSS effectors controlled by MxiE	Shigella	Shigella flexneri	21.858	55.290	14.610	33.826
IdeR	Mycobacterium	Mycobacterium tuberculosis	60.592	6.648	5.038	48.825
O-antigen	Yersinia	Yersinia enterocolitica subsp.	12.210	42.876	40.174	24.897

Capsule	Klebsiella	Klebsiella pneumoniae subsp.	16.233	42.627	18.216	42.278
Alginate	Pseudomonas	Pseudomonas aeruginosa	8.786	52.490	17.708	39.945
Pantothenate synthesis	Mycobacterium	Mycobacterium gilvum	59.412	3.952	4.154	46.621
Fibronectin-binding protein	Mycobacterium	Mycobacterium tuberculosis	14.558	29.161	13.032	56.288
Polar flagella	Aeromonas	Aeromonas hydrophila	8.530	52.909	21.178	27.828
Exopolysaccharide	Haemophilus	Haemophilus ducreyi	14.979	39.837	32.591	18.679
Flagella	Bartonella	Bartonella bacilliformis	13.565	47.515	22.836	22.046
AcrAB	Klebsiella	Klebsiella pneumoniae subsp.	3.258	57.639	20.234	22.097
AhpC	Mycobacterium	Mycobacterium smegmatis str.	22.230	29.152	24.478	26.817
Vi antigen	Salmonella	Salmonella enterica subsp.	12.826	45.864	23.484	14.414
Polar flagella	Aeromonas	Aeromonas hydrophila subsp.	3.048	41.314	29.534	14.065
DevR/S	Mycobacterium	Mycobacterium tuberculosis str.	37.060	1.011	3.495	40.691
Tap type IV pili	Aeromonas	Aeromonas hydrophila subsp.	3.141	40.219	26.880	9.583
Cyp125	Mycobacterium	Mycobacterium smegmatis str.	28.393	0.938	7.417	41.810

Table S8 Effects of soil properties on soil microbial communities, antibiotics resistance genes (ARGs) and virulence factors (VFs).

Type		RDA1	RDA2	r^2	p-value
Microbial community	AN	-0.999938936	-0.011051023	0.830	0.002
	AK	0.312011187	0.950078428	0.085	0.656
	AP	-0.996307021	0.085862218	0.666	0.017
	NH_4^+-N	-0.291391901	-0.956603763	0.228	0.336
	SOC	0.514757095	-0.857336068	0.245	0.276
	pH	-0.729267547	-0.684228649	0.160	0.472
	EC	-0.978518021	0.206161302	0.591	0.021
	salinity	-0.978091446	0.208175705	0.594	0.021
AEGs	AN	-0.928713585	-0.370797893	0.974	0.001
	AK	-0.017307775	-0.999850209	0.799	0.004
	AP	-0.935292891	0.353874565	0.716	0.004
	NH_4^+-N	-0.329249984	0.944242791	0.026	0.870
	SOC	0.466298012	0.884627698	0.282	0.224
	pH	-0.993330356	-0.115303091	0.050	0.789
	EC	-0.914593835	-0.404373735	0.664	0.011
	salinity	-0.90715882	-0.420788397	0.678	0.011
VFs	AN	-0.99998036	-0.006267279	0.908	0.001
	AK	-0.326778083	-0.945101097	0.868	0.001
	AP	-0.888114114	0.45962302	0.752	0.004
	NH_4^+-N	0.431166679	0.902272295	0.239	0.303
	SOC	0.680820139	0.732450639	0.637	0.013
	pH	-0.014593892	0.999893503	0.102	0.627
	EC	-0.976550856	-0.215286846	0.873	0.002
	salinity	-0.973804025	-0.227388918	0.888	0.001

AN, alkaline hydrolyzable nitrogen; AK, available potassium; AP, available phosphorus; NH_4^+-N, ammonium nitrogen; SOC, soil organic carbon; pH, soil pH; EC, electrical conductivity; Salinity, soil salinity.

Table S9 Topological parameters ofMicrobial-ARG-VF interaction networks across four rice seedling cultivation soils.

Typle	Node Name	Degree	Degree Centrality	Closeness Centrality	Betweenness Centrality
ARGs vs VFs	rpoB2	5	0.128	0.398	0.007
	Nitrate reductase	14	0.359	0.557	0.051
	Type IV pili	12	0.308	0.527	0.037
	Urease	13	0.333	0.542	0.043
	Flagella	5	0.128	0.375	0.012
	AdeFGH efflux pump	5	0.128	0.424	0.006
	vanSO	14	0.359	0.527	0.016
	EF-Tu	15	0.385	0.557	0.018
	Hsp60	15	0.385	0.557	0.018
	MOMP	16	0.410	0.591	0.031
	Glutamine synthesis	15	0.385	0.557	0.018
	ClpC	9	0.231	0.476	0.021
	Proteasome-associated proteins	15	0.385	0.557	0.018
	GPL locus	16	0.410	0.591	0.031
	Pyrimidine biosynthesis	16	0.410	0.591	0.029
	Isocitrate lyase	11	0.282	0.500	0.030
	Trehalose-recycling ABC transporter	15	0.385	0.557	0.018
	Streptococcal enolase	15	0.385	0.557	0.018
	ClpP	15	0.385	0.574	0.021
	RelA	15	0.385	0.557	0.018
	Heme biosynthesis	16	0.410	0.591	0.031
	MuxB	8	0.205	0.433	0.003
	mtrA	17	0.436	0.609	0.031
	KatAB	11	0.282	0.500	0.007
	Streptomyces rishiriensis parY mutant conferring resistance to aminocoumarin	17	0.436	0.609	0.032
	vanRO	15	0.385	0.542	0.019
	Bifidobacterium adolescentis rpoB mutants conferring resistance to rifampicin	16	0.410	0.574	0.025
	vanHO	16	0.410	0.574	0.025
	MexF	8	0.205	0.464	0.022
	tetA(58)	14	0.359	0.527	0.016
	novA	16	0.410	0.591	0.025
	vanXO	17	0.436	0.591	0.052
	iri	18	0.462	0.629	0.040
	oleC	17	0.436	0.609	0.031
	rphA	10	0.256	0.500	0.036
	vanO	16	0.410	0.574	0.025

	Bifidobacterium ileS conferring resistance to mupirocin	3	0.077	0.382	0.006
	tetB(58)	14	0.359	0.527	0.016
	vanJ	18	0.462	0.629	0.040
	adeF	5	0.128	0.398	0.007
	g__unclassified_p__Chloroflexi|p__ Chloroflexi	11	0.282	0.476	0.024
	MOMP	14	0.359	0.557	0.022
	Nitrate reductase	10	0.256	0.500	0.010
	GPL locus	15	0.385	0.574	0.026
	Pyrimidine biosynthesis	15	0.385	0.574	0.025
	Streptococcal enolase	17	0.436	0.609	0.044
	Type IV pili	10	0.256	0.500	0.010
	Urease	10	0.256	0.500	0.010
	ClpP	14	0.359	0.557	0.022
	Flagella	3	0.077	0.424	0.001
	Heme biosynthesis	15	0.385	0.574	0.026
	AdeFGH efflux pump	4	0.103	0.415	0.002
	g__Actinocatenispora|p__ Actinobacteria	17	0.436	0.609	0.047
	EF-Tu	11	0.282	0.513	0.013
	Hsp60	13	0.333	0.542	0.018
Microbial communities vs VFs	KatAB	16	0.410	0.591	0.163
	Glutamine synthesis	14	0.359	0.557	0.021
	Proteasome-associated proteins	14	0.359	0.557	0.021
	Trehalose-recycling ABC transporter	14	0.359	0.557	0.022
	RelA	15	0.385	0.574	0.025
	g__Streptomyces|p__Actinobacteria	13	0.333	0.542	0.020
	ClpC	7	0.179	0.453	0.004
	Isocitrate lyase	10	0.256	0.488	0.007
	g__unclassified_c__Alphaproteobacteria|p __Proteobacteria	7	0.179	0.433	0.003
	g__Amycolatopsis|p__Actinobacteria	13	0.333	0.542	0.020
	g__unclassified_p__Proteobacteria|p __Proteobacteria	10	0.256	0.464	0.020
	g__Pseudonocardia|p__Actinobacteria	16	0.410	0.591	0.029
	g__unclassified_o__Streptosporangiales|p __Actinobacteria	17	0.436	0.609	0.034
	g__Actinomadura|p__Actinobacteria	18	0.462	0.629	0.043
	g__unclassified_c__Actinomycetia|p __Actinobacteria	1	0.026	0.375	0.000
	g__Microlunatus|p__Actinobacteria	14	0.359	0.557	0.023
	g__Alloacidobacterium|p__Acidobacteria	1	0.026	0.375	0.000

	g__Kribbella|p__Actinobacteria	17	0.436	0.609	0.034
	g__unclassified_p__Acidobacteria|p __Acidobacteria	17	0.436	0.609	0.047
	g__Reticulibacter|p__Chloroflexi	1	0.026	0.375	0.000
	g__unclassified_o__Hyphomicrobiales|p __Proteobacteria	8	0.205	0.443	0.004
	g__unclassified_o__Pseudonocardiales|p __Actinobacteria	17	0.436	0.609	0.034
	g__unclassified_c__Acidimicrobiia|p __Actinobacteria	18	0.462	0.629	0.043
	g__Nocardia|p__Actinobacteria	11	0.282	0.513	0.015
	g__Nocardioides|p__Actinobacteria	14	0.359	0.557	0.049
	g__unclassified_p__Chloroflexi|p __Chloroflexi	12	0.308	0.453	0.044
	rpoB2|Multidrug	3	0.077	0.406	0.001
	mtrA|Multidrug	15	0.385	0.557	0.031
	Bifidobacterium adolescentis rpoB mutants conferring resistance to rifampicin|Multidrug	14	0.359	0.527	0.022
	vanHO|Glycopeptide	14	0.359	0.527	0.022
	MexF|Multidrug	7	0.179	0.443	0.006
	vanXO|Glycopeptide	14	0.359	0.527	0.022
	iri|Rifamycin	14	0.359	0.542	0.022
	oleC|MLS	15	0.385	0.557	0.031
	rphA|Rifamycin	10	0.256	0.476	0.011
	vanO|Glycopeptide	14	0.359	0.527	0.022
Microbial communities vs ARGs	vanJ|Glycopeptide	15	0.385	0.557	0.031
	adeF|Multidrug	2	0.051	0.320	0.000
	g__Actinocatenispora|p__Actinobacteria	16	0.410	0.591	0.037
	vanSO|Glycopeptide	11	0.282	0.500	0.025
	Streptomyces rishiriensis parY mutant conferring resistance to aminocoumarin|Aminocoumarin	15	0.385	0.557	0.043
	vanRO|Glycopeptide	12	0.308	0.513	0.029
	tetA(58)|Tetracycline	14	0.359	0.542	0.038
	novA|Aminocoumarin	16	0.410	0.574	0.163
	tetB(58)|Tetracycline	14	0.359	0.542	0.038
	g__Streptomyces|p__Actinobacteria	12	0.308	0.527	0.016
	g__unclassified_c__Alphaproteobacteria|p __Proteobacteria	6	0.154	0.406	0.022
	MuxB|Multidrug	3	0.077	0.315	0.004
	g__Amycolatopsis|p__Actinobacteria	12	0.308	0.527	0.017

g__unclassified_p__Proteobacteria|p __Proteobacteria	11	0.282	0.443	0.038
g__Pseudonocardia|p__Actinobacteria	16	0.410	0.591	0.037
g__unclassified_o__Streptosporangiales|p __Actinobacteria	16	0.410	0.591	0.037
g__Actinomadura|p__Actinobacteria	15	0.385	0.574	0.029
g__unclassified_c__Actinomycetia|p __Actinobacteria	1	0.026	0.368	0.000
g__Microlunatus|p__Actinobacteria	12	0.308	0.527	0.016
g__Alloacidobacterium|p__Acidobacteria	2	0.051	0.390	0.008
g__Kribbella|p__Actinobacteria	15	0.385	0.574	0.029
g__unclassified_p__Acidobacteria|p __Acidobacteria	17	0.436	0.609	0.066
g__Reticulibacter|p__Chloroflexi	1	0.026	0.368	0.000
g__unclassified_o__Hyphomicrobiales|p __Proteobacteria	6	0.154	0.406	0.022
g__unclassified_o__Pseudonocardiales|p __Actinobacteria	16	0.410	0.591	0.037
g__unclassified_c__Acidimicrobiia|p __Actinobacteria	15	0.385	0.574	0.029
g__Nocardia|p__Actinobacteria	11	0.282	0.513	0.013
g__Nocardioides|p__Actinobacteria	11	0.282	0.513	0.064
Bifidobacterium ileS conferring resistance to mupirocin|Mupirocin	1	0.026	0.342	0.000
